# Supplementary material for: Unstable Inheritance of 45S rRNA Genes in Arabidopsis thaliana
Source: G3 (Bethesda). 2017 Feb 10;7(4):1201–9. doi: 10.1534/g3.117.040204 (PMC5386868; doi:10.1534/g3.117.040204)
Supplement: Supplementary file 3 [file 1201FileS3.docx]

## **Supplemental Material**


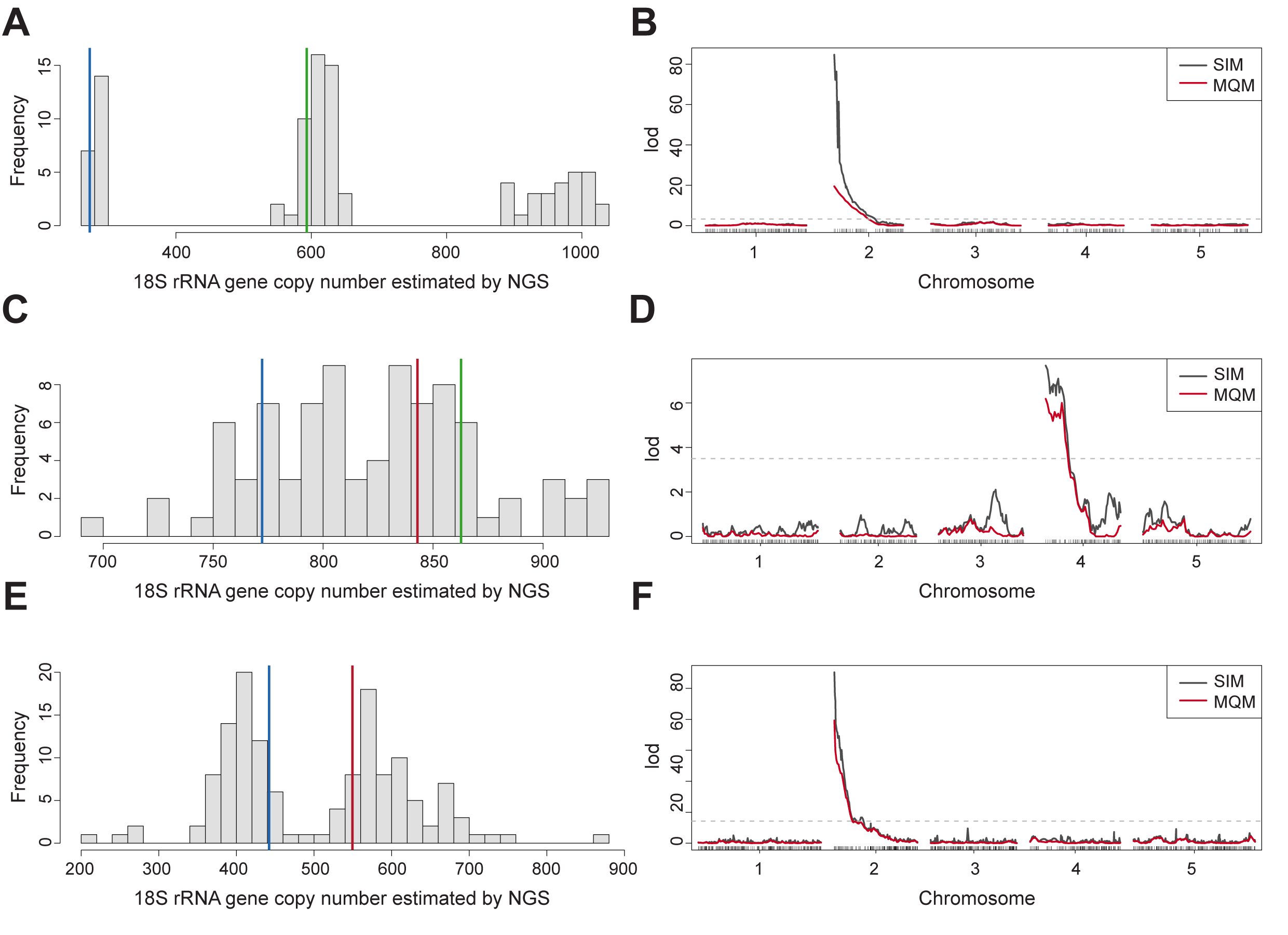


**Figure S1. rRNA gene copy number variation in F2 progenies and a RIL population.**

(A) The distribution of 18S rRNA gene copy number estimated by NGS in an F2 population of 92 individuals derived from the cross Ull1-1 (8426) x TDr-7 (6193). Blue and green vertical lines represent phenotypic values of accession TDr-7 and an F1 individual, respectively (data for Ull1-1 are missing). (B) QTL mapping of 18S rRNA gene copy number in the same F2 population described in (A). (C) The distribution of 18S rRNA gene copy number estimated by NGS in an F2 population of 84 individuals derived from the cross T460 (6106) x Omn-5 (6071). Blue, green and red vertical lines represent phenotypic values of accession Omn-5, an F1 individual and accession T460, respectively. Note that the difference between the parental lines is small relative to the measurement error, and that this likely explains the “transgressive” value of the F1 individual. (D) QTL mapping of 18S rRNA gene copy number in the same F2 population described in (C). (E) The distribution of 18S rRNA gene copy number estimated by NGS in a RIL population of 134 individuals derived from cross Cvi-0 (6911) x Ler-0 (7213). Blue and red vertical lines represent phenotypic values of parental accessions Cvi-0 and Ler-0, respectively. (F) QTL mapping of rRNA gene copy number in the same RIL population described in (E).


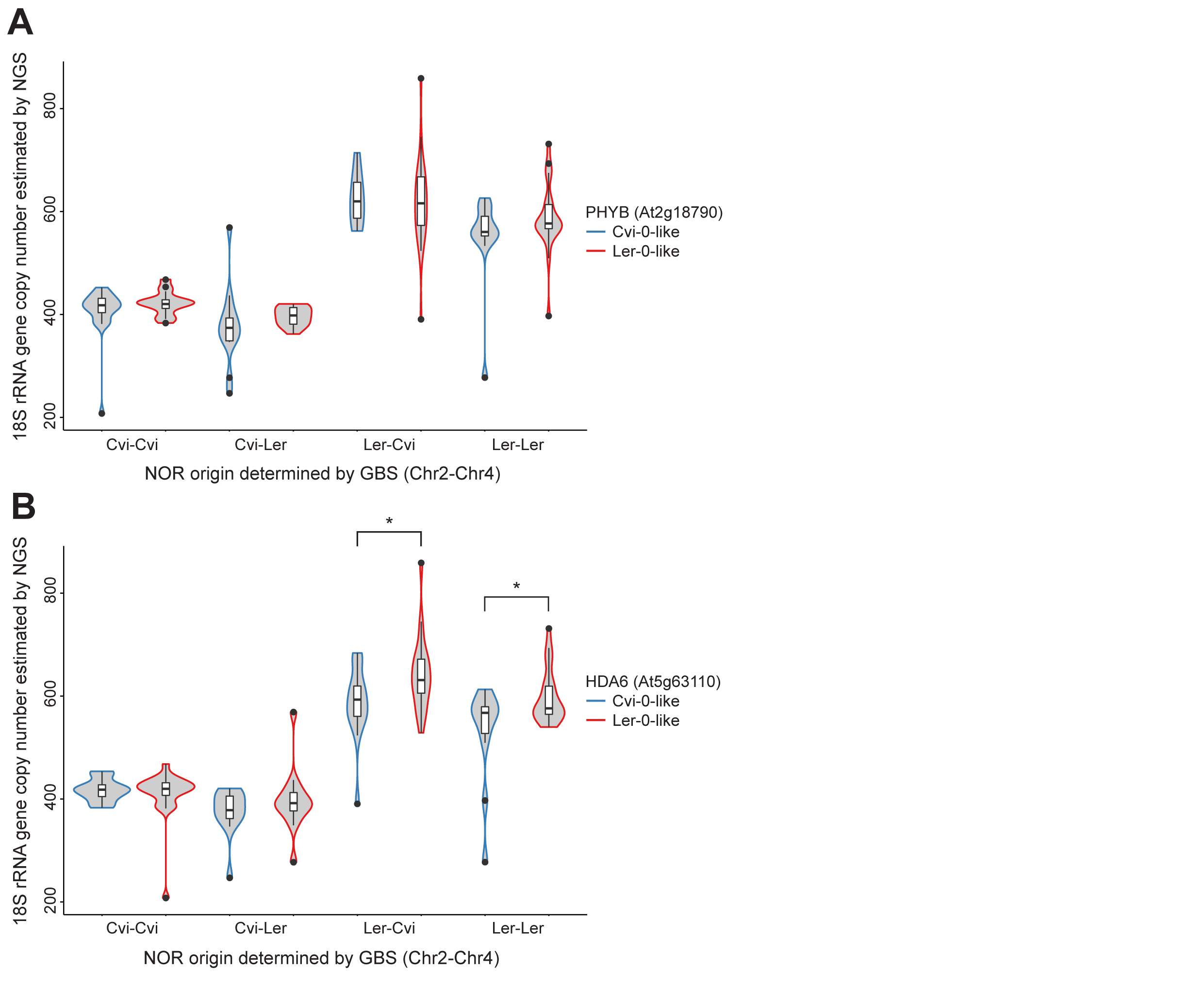


**Figure S2. The effect of Cvi-0 and Ler-0 alleles at *PHYB* and *HDA6* genes in rRNA gene copy number.**

(A) 18S rRNA gene copy number in the Cvi-0 x Ler-0 RIL population estimated by NGS split — first — by NOR parental identity and — second — by the allele inherited at the *PHYB* locus as determined by GBS. Blue and red contours of the violin plots indicate alleles Cvi-0-like and Ler-0-like at the *PHYB* gene (At2g18790), respectively. P-values for the effect in rRNA gene copy number of parental NORs (NOR2-NOR4) Cvi-Cvi, Cvi-Ler, Ler-Cvi and Ler-Ler as a function of *PHYB* are 0.486, 0.897, 0.495 and 0.450, respectively. (B) 18S rRNA gene copy number in the Cvi-0 x Ler-0 RIL population estimated by NGS split — first — by NOR parental identity and — second — by the allele inherited at the *HDA6* locus as determined by GBS. Blue and red contours of the violin plots indicate alleles Cvi-0-like and Ler-0-like at the *HDA6* gene (At5g63110), respectively. P-values for the effect in rRNA gene copy number of parental NORs (NOR2-NOR4) Cvi-Cvi, Cvi-Ler, Ler-Cvi and Ler-Ler as a function of *PHYB* are 0.7038, 0.3731, 0.0298 and 0.0125, respectively. The asterisk (*) represent a p-value < 0.05.


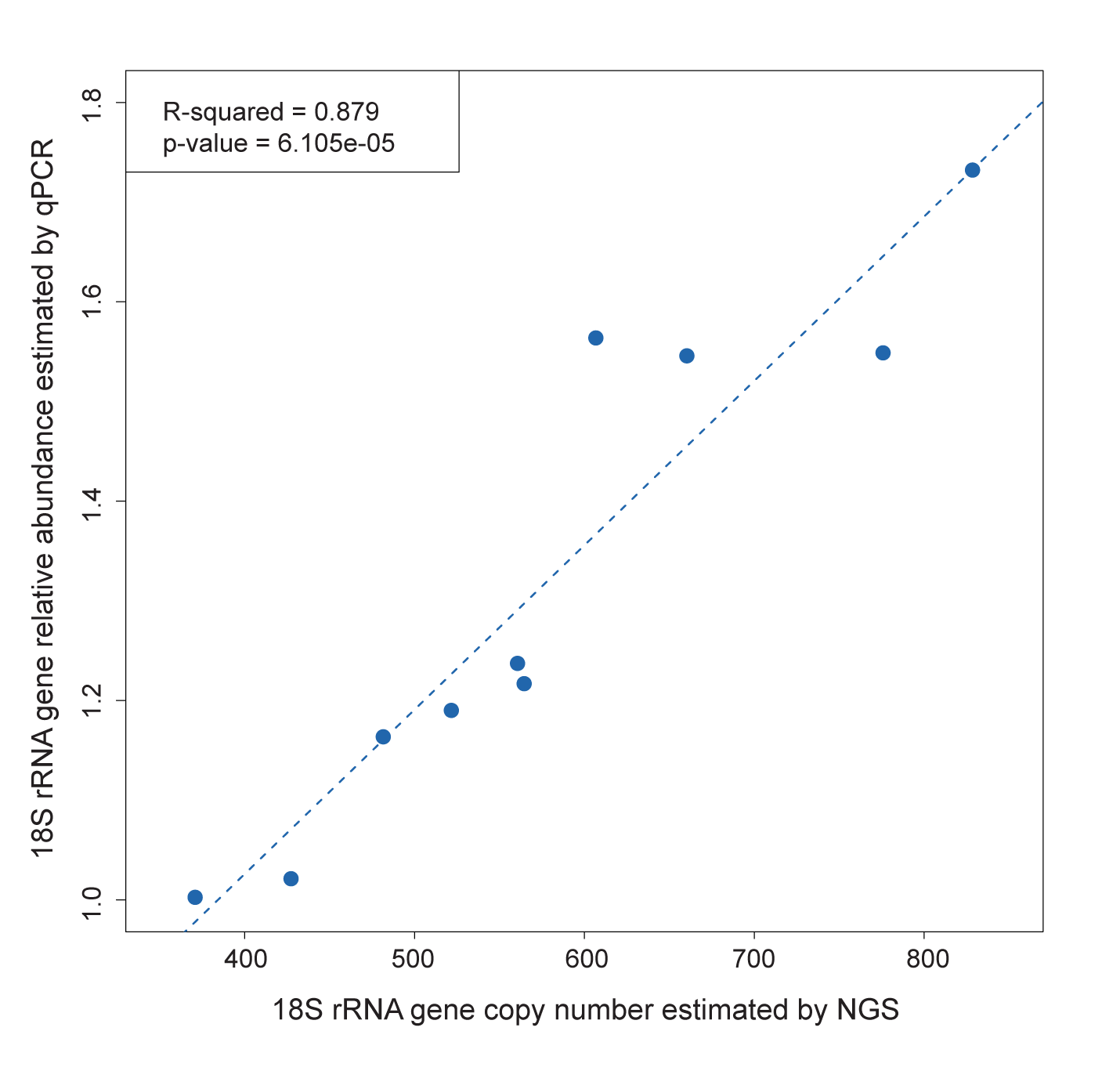


**Figure S3. Correlation between two estimators of 18 rRNA gene copy number for the MA lines**

Correlation between two estimators of 18 rRNA gene copy number for the MA lines: Next generation sequencing (NGS) and quantitative PCR (qPCR).

| **Table S1. Nucleolar association of NOR2 and NOR4 in two accessions.**   \|  \| **NORs associated with the nucleolus** \| \| \| \| \| \| --- \| --- \| --- \| --- \| --- \| --- \| \| **Accession name**  **(n = number of nuclei)** \| **NOR2 2x**  n (%) \| **NOR2 2x &**  **NOR4 1x**  n (%) \| **NOR2 2x &**  **NOR4 2x**  n (%) \| **NOR2 1x &**  **NOR4 2x**  n (%) \| **NOR4 2x**  n (%) \| \| Ale-Stenar-64-24 (n = 19) \| 0 \| 0 \| 3 (16%) \| 8 (42%) \| 8 (42%) \| \| TRÄ-01 (n = 25) \| 10 (40%) \| 14 (56%) \| 1 (4%) \| 0 \| 0 \|   Relative frequency of root-tip nuclei with a particular NOR configuration relative to its close proximity to the nucleolus for the parental accessions Ale-Stenar-64-24 (1002) and TRÄ-01 (6244). n is the number of nuclei in each category. |
| --- | --- | --- | --- | --- | --- | --- | --- | --- | --- | --- | --- | --- | --- | --- | --- | --- | --- | --- | --- | --- | --- | --- | --- | --- |

**File S1. 45S rRNA gene reference.**

**File S2. Genetic maps of F2s and RILs, phenotypes of F2s, RILs, MAGIC and MA lines.**
